# Notes and News

**Published:** 1888-11-17

**Authors:** 


### NOTES AND NEWS.

COLLECTED AND COMMUNICATED.

THE Cremation Society's new chapel, waiting-rooms, and lodge in the grounds at Woking are approaching completion. Fifty-one bodies have already been cremated. The Scottish Burial Reform and Cremation Society are arranging for the immediate erection of buildings, including a chapel and columbarium, on a picturesque site adjoining the Glasgow Cathedral.

THE epidemic of diphtheria is creating a scare, but Walthamstow has vindicated itself of being badly drained. Dr. Airy reports to the Local Government Board that the annual death-rate from diphtheria for the whole of West Ham during the last ten years was only 0·1 per thousand. There can be little doubt that the late sewage floods had something to do with the present outbreak.

THE first report of the new Cottage Hospital at Cromer has been issued, and will be read with interest by those who have lent a helping hand to this undertaking. Thirty patients have already benefited by the beautiful and commodious building erected by Mr. Collinson, and, in spite of the increased expenditure, the funds are in a satisfactory state, and hospital affairs are flourishing at Cromer, if nowhere else.

NEARLY every winter various sewing societies meet in towns and villages, to work for our hospitals. The excellence of this plan is only thoroughly appreciated by the sisters and nurses of wards, who have dismissed on a cold day semi-convalescents who are insufficiently clad. The Glasgow Royal Infirmary Dorcas Society has just had its first meeting to obviate such unhappiness, and its example might well be followed in many circles.

A SPECIAL meeting of the Managing Committee and subscribers of the Hull Royal Infirmary was held in the board-room of the infirmary last week, for the purpose of presenting Mr. Henry Simpson, late Chairman of the Managing Committee, with his portrait in recognition of the many valuable services rendered by him to the infirmary. The portrait, which was painted by Mr. J. R. West, was a faithful likeness. Mr. Simpson requested the Committee to accept the portrait, to hang in the board-room.

THE half-yearly meeting of the Committee of the British Home for Incurables was held on November 8th, at the Cannon-street Hotel, under the presidency of Mr. Francis A. Bevan. Sixteen candidates were elected instead of the usual eleven, the extra five being in commemoration of the silver wedding of the Prince and Princess of Wales. It was announced that the Board had decided to build a new home on the present site at a cost of £15,000, with every modern improvement for the care of incurable sufferers. Already two ladies had promised £1,000 to endow a bed in the new home when finished, and he trusted that the public would come forward with liberal gifts to the rebuilding fund.

THE yellow fever in Jacksonville is decreasing. There are only about twenty cases now in Jacksonville, and a few isolated cases in other towns, where there is no fear of its spreading. Since the outbreak of the epidemic there have been 4,240 persons seized with the fever, and 359 have died. This is a much smaller percentage of deaths than occurred during the former outbreak, and there can be no doubt that in time the dreaded yellow fever will be conquered by science.

WE have received from Dr. Alford the annual report of the Taunton Sanitary Hospital. The hospital has been of great service in checking epidemics and affording attendance and comforts to the patients who have sought its shelter. Of a hospital of this sort it is satisfactory to notice a decrease in the number of patients—63 patients were treated this year, against 93 treated last year. Of these cases 7 died—5 from diphtheria and 2 from enteric fever. The other 55 were discharged cured, and 1 patient remained in hospital.

DONCASTER Infirmary issues a splendid annual report. The governors have the gratification of finding that the income responds to the increasing needs of the patients: the in-patients numbered 200, and the dispensary patients 2,361, both being an increase on the previous year, and more especially so with regard to the latter. The annual subscriptions amounted to £666, and the "Infirmary Saturday" collections to £256, the annual appeal made on "Infirmary Sunday" reaching, within a few shillings, the contributions of last year.

THE discussion which has lately taken place on the Metropolitan Asylums Board on the subject of compensation in the case of an owner of property near the Winchmore Hill Hospital for infectious diseases once more draws attention to the hardships which the proximity of such institutions sometimes inflict. In the case referred to it was shown that the effect of erecting the hospital in question had been to drive away the tenants of a number of houses. Amongst others, a widow had suffered to the extent of £200. It was proposed to grant her some compensation, but the motion was negatived.

THE seventy-fourth anniversary of the Derbyshire General Infirmary was celebrated on November 9th. Shortly before eleven o'clock, the Mayor and many of the Corporation met at the Town Hall, and went in procession to All Saints' Church, where a special sermon was preached by the Dean of Lichfield. The collection amounted to £130. Afterwards the annual meeting was held in the Board Room of the Infirmary, when a most satisfactory report was read. For the first time this meeting was attended by many of the leading ladies of the town (including Lady Wilmot), who were warmly welcomed by the president. Mr. H. J. Wood was nominated president for the coming year; and a vote of condolence was passed with the members of the family of the late Dr. Taylor, for many years a valued medical officer of the institution.

THE question of how to establish a provident dispensary on a sound financial basis is exercising the minds of the governers of the Brighton, Hove, and Preston Institution. There can be no doubt that the greatest amount of medical service at the least cost is given by a well-managed dispensary. At Brighton, however, they have been running their institution at an annual loss of two or three hundred pounds, and, as larger premises have just been secured in Sackville Road, it became necessary to remedy this defect, which might otherwise have got beyond control. The following motions were therefore approved: 1. That a registration fee of 6d. be paid by each patient on presenting a letter of recommendation. 2. That the powers of investment of the funds of the institution be enlarged.

The following vacancies are announced this week, the amount of salary in each case being given after the name of the institution :—

**Resident Medical Officers.**

Dover Hospital and Dispensary. House Surgeon.—£100, board, etc.
Bradford Infirmary and Dispensary. House Physician.—£100, board, etc.
Grantham Friendly and Trades Societies. Medical Officer.—£150, residence, etc.
Rainhill Asylum. Medical Superintendent.—£1,000.
Newcastle-upon-Tyne Borough Lunatic Asylum. Superintendent. —£450, residence, etc.

**Non-resident Medical Officers.**

Royal South Hants Infirmary. Surgeon and Assistant Surgeon.
Paddington Green Children's Hospital. Honorary Physician.
Croydon Union District Medical Officer —£120.
Free Hospital for Children of the Poor, Kingsholm, Gloucester. Surgeon

The constant disagreements at the Mercers' Hospital, Dublin, have ended in such a sad state of things that it is not to participate in the fund collected last Sunday. A governor of the hospital and Dr. Knight have both written to the Dublin *Irish Times* trying to establish a better feeling between the hospital and the fund, and it is to be hoped that in the end all will be amicably settled, for the public are getting tired of the continuous and utterly unnecessary quarrelling.

Hospital Sunday has come to be such a settled institution in every town and village that it scarcely seems possible to realise that Ballymena knows it not. There is a splendid little hospital at Ballymena which treats some eighty in-patients yearly, and yet the appeal of the committee for a Hospital Sunday has met with no response. In every town there are plenty of people with charitable inclinations, who cannot afford to become regular subscribers to an institution, perhaps because their income fluctuates, perhaps because they do not care to put a small sum opposite their name. These are the people whose shillings and pence mount up when churches grant them a chance of giving quietly and secretly.

During the year building operations with the new Tynemouth Infirmary have been progressing favourably. The building was commenced in April last, and it is anticipated that the hospital will be ready for occupation in June next. The building fund may be considered fairly satisfactory. The total amount subscribed to date is £2,646 12*s*., leaving a balance of £353 8*s*. yet to be got to cover the contract price, £3,000. It may be stated that £3,000 does not cover the professional and incidental expenses. The Building Committee are exceedingly anxious that the hospital should be opened free of debt, and strenuous efforts will be put forth to accomplish that desirable end. The workmen of Tynemouth afford systematic and continued support to the Infirmary, greatly to the pleasure of the committee.

The Indian ladies of Lahore have presented a most affectionate and touching address to the Countess Dufferin, thanking her for her efforts on their behalf in connexion with the National Association of Medical Aid. The address concludes: "We are sure that when the history of the period during which your Excellency graced this country with your presence comes to be written, your Excellency will figure in its pages as one of the noblest and greatest benefactresses India ever has had. We are aware that the work your Excellency has done for your poor and helpless Indian sisters is so permanent and enduring that your Excellency's name will always be remembered by them and their future generations with sentiments of the deepest veneration and esteem. In taking leave of your Excellency we most earnestly pray that, May the Great Almighty confer upon you and yours every blessing, happiness, and prosperity."

Dr. Richard Greene, Superintendent of the County Asylum, Berry Wood, near Northampton, has been elected a Fellow of the Royal College of Physicians, Edinburgh. We congratulate Dr. Greene on the honour which has been conferred upon him. Dr. Greene is generally recognised as one of the ablest and most experienced asylum superintendents in this country. Indeed, we do but give expression to the general feeling in declaring that his public services and devotion have marked him out for still greater promotion in his profession in the near future.

Dr. E. A. Birch, Surgeon-Superintendent of the Presidency General Hospital, Calcutta, has written a letter to the *Times*, contending that the consensus of opinion is in favour of maintaining the Netley Medical School. Dr. Birch maintains that a medical officer should not go to India until he is fully qualified and fit for duty anywhere. Otherwise, when he arrives in a trying climate, with a strange language and utterly new ways of life to learn, he will break down. During his first years in India, the young surgeon should be attached to a Presidency Civil Hospital, and go through a course of military hygiene; but physical arguments against forming an Army Medical School in India are almost overwhelming.

An important discussion took place at the last meeting of the Stockport Infirmary Committee, on the religious ministrations to the patients. The question was raised by the Rev. J. Robinson, whether the resolution that clergymen and ministers of all denominations should be permitted to enter the building for personal and ministerial visiting meant that a minister might enter a ward in which were patients of all denominations, and minister in accordance with the practices of his own body; or whether he was simply to minister at the bedside of those belonging to his own sect. This question was brought forward because Father Robinson had seen a patient cover her head with the clothes so as not to hear a service which was being held, and which she did not believe in. After a discussion, in which Dr. Raynor spoke very strongly, Father Robinson's proposal that ministrations should be confined to the bedside was negatived. We are sorry that the committee so decided, because experience shows that if every minister of religion who enters a hospital ward to visit a member of his congregation is to understand that he is at liberty not only to attend to him, but to conduct a service to include all patients of whatever religion they may be, and whether they wish to take part in it or not, it is perfectly certain that the Stockport Infirmary will soon cease to be of service to the sick. Under such regulations the whole establishment must practically be reduced to a state of disorder and discomfort, which we should have thought the common sense and good feelings of any body of gentlemen must have spared the hospital patients in their charge. It is almost, if not quite, the invariable custom in English hospitals to put a large screen round the bed whilst a clergyman is ministering to the sick member of his flock. Let Stockport be warned in time, and decide to follow the general practice, or she will soon repent it. We will suppose a case in proof of this. A Roman Catholic priest calls at the hospital to administer extreme unction in one of the large wards to a dying member of his flock. No screen is provided, and, having robed in the board-room, he ascends the staircase with his acolytes in full canonicals with candles lighted. The ward door is thrown open, the procession enters, passes down the middle of the ward, and stops by the bedside of the dying. The last rites of the Church are there and then publicly administered to the poor sufferer, after which a general service is conducted in the ward, and each patient is visited in turn. Such is the scene which the Stockport committee, as we understand, have not only sanctioned, but invited. Will they really permit it in practice?

